# The Empathy for Pain Stimuli System (EPSS): Development and preliminary validation

**DOI:** 10.3758/s13428-023-02087-4

**Published:** 2023-03-02

**Authors:** Jing Meng, Yanting Li, Longli Luo, Lingxiao Li, Jin Jiang, Xiaocui Liu, Lin Shen

**Affiliations:** 1 Key Laboratory of Applied Psychology, Chongqing Normal University, Chongqing, China; 2 Research Center for Brain and Cognitive Science, School of Educational Sciences, Chongqing Normal University, Chongqing, China; 3 School of Mathematical Sciences, Chongqing Normal University, Chongqing, China

**Keywords:** Empathy for pain, Database, Picture, Voice, Video

## Abstract

We present the Empathy for Pain Stimuli System (EPSS): a large-scale database of stimuli for studying people’s empathy for pain. The EPSS comprises five sub-databases. First, the Empathy for Limb Pain Picture Database (EPSS-Limb) provides 68 painful and 68 non-painful limb pictures, exhibiting people’s limbs in painful and non-painful situations, respectively. Second, the Empathy for Face Pain Picture Database (EPSS-Face) provides 80 painful and 80 non-painful pictures of people’s faces being penetrated by a syringe or touched by a Q-tip. Third, the Empathy for Voice Pain Database (EPSS-Voice) provides 30 painful and 30 non-painful voices exhibiting either short vocal cries of pain or neutral interjections. Fourth, the Empathy for Action Pain Video Database (EPSS-Action_Video) provides 239 painful and 239 non-painful videos of whole-body actions. Finally, the Empathy for Action Pain Picture Database (EPSS-Action_Picture) provides 239 painful and 239 non-painful pictures of whole-body actions. To validate the stimuli in the EPSS, participants evaluated the stimuli using four different scales, rating pain intensity, affective valence, arousal, and dominance. The EPSS is available to download for free at https://osf.io/muyah/?view_only=33ecf6c574cc4e2bbbaee775b299c6c1.

**Supplementary Information:**

The online version contains supplementary material available at 10.3758/s13428-023-02087-4.

## Introduction

Empathy for pain is a multidimensional psychological process that allows people to recognize and share others’ feelings of pain (Cui et al., 2017a; Li et al., 2019; Peng et al., 2021; Ren et al., 2020; Zhang et al., 2022). Due to its specific cognitive and neural mechanisms, researchers have paid significant attention to empathy for pain, making it a popular topic in empathy studies. Researchers often use experiments with stimuli representing other people’s pain to explore the potential mechanisms behind empathy for pain.

Over the last few decades, the wealth of experimental research regarding people’s empathy for pain has been supplemented by several resources for testing. When individuals observe, hear, or imagine other people in painful situations, they can experience their affective and cognitive states in their minds (Penga et al., 2019). Typically, previous studies of empathy for pain have used visual or auditory stimuli.

According to a meta-analysis of 40 studies on ERP investigations of pain empathy (Coll, 2018), 90% (36 studies) of visual studies of empathy for pain used pictures (33 studies, 82.5%) or short videos (three studies, 7.5%) depicting human limbs (such as hands or feet) exposed to harmful stimuli (for example, a hand cut by a knife or foot penetrated by a syringe). These images were placed alongside images of limbs subject to non-harmful stimuli (for example, a hand using a knife to cut vegetables or a foot being touched by a Q-tip). These images have generally been similar to those initially used by Jackson et al. (Jackson et al., 2005). Compared with non-painful limb stimuli, painful limb stimuli typically elicit shorter reaction times (RTs) (Fabi & Leuthold, 2018; Wang et al., 2016) and less pleasure (Gonzalez-Liencres et al., 2016). Painful stimuli also activate the anterior cingulate cortex and several other areas (Gu & Han, 2007) and provoke larger P3 and long-latency positive component event-related potential (ERPs) amplitudes (Cheng et al., 2014; Cui et al., 2017b; Fan & Han, 2008; Meng et al., 2013; Meng et al., 2019b).

Approximately 7.5% (three of 40 studies, according to Coll, 2018) of visual studies on empathy for pain used images depicting faces of individuals pricked by a needle (painful faces) or gently touched by a Q-tip (non-painful faces). Studies revealed that painful faces induced longer RTs (Li et al., 2020), larger N2 and P3 amplitudes (Li et al., 2020; Meng et al., 2020a), and more activity in the anterior cingulate cortex (Han et al., 2009) than non-painful faces did.

Most studies of empathy for pain have focused on the visual modality, but recognizing pain from others’ voices is the auditory equivalent of visual pain recognition. The Montreal Affective Voices database (Belin et al., 2008) included ten nonverbal outcries of voices in pain and ten neutral voices recorded by ten actors (five women). However, the duration of these voices varied from 432 to 1528 ms. Previous studies manipulated these voices to achieve a duration of 700 ms and a mean intensity of 70 dB (Liu et al., 2019; Meng et al., 2019a, 2019b; Meng, Li, & Shen, 2020b). This allowed them to explore the behavioral and neural mechanisms behind empathy for auditory pain. Painful voices elicit higher accuracies (ACCs) and shorter RTs (Meng et al., 2019b), greater judgments of the intensity of pain, more negative emotional reactions, and larger P2 amplitudes (Meng et al., 2019a) than neutral voices.

However, the results of studies using different stimuli of others’ pain have not been consistent (Josiane et al., 2019). This may be because some of these studies did not control the luminance, contrast, and color of the painful and non-painful visual stimuli or the duration and intensity of the painful and non-painful auditory stimuli. In addition, some of these stimuli were not evaluated well. They were not selected based on basic emotional dimensions, such as pain intensity, affective valence, arousal, and dominance. Moreover, although understanding others’ pain in real life involves interpreting various cues, including postures and actions, to our knowledge, only a few studies (e.g., Li et al., 2022) have used human postures and actions to investigate empathy for pain. Using the whole body may have better ecological validity than only using part of the body.

The present study aimed to make available a richly varied, well-validated, and open-access stimuli database for researchers examining empathy for pain. This is the Empathy for Pain Stimuli System (EPSS). The EPSS consists of five sub-databases: (1) the Empathy for Limb Pain Picture Database (EPSS-Limb), (2) the Empathy for Face Pain Picture Database (EPSS-Face), (3) the Empathy for Voice Pain Database (EPSS-Voice), (4) the Empathy for Action Pain Video Database (EPSS-Action_Video), (5) the Empathy for Action Pain Picture Database (EPSS-Action_Picture). Each database consists of various painful stimuli and corresponding non-painful stimuli. All the stimuli were recorded by actors or revised from stimuli previously validated in other published studies. The five sub-databases were validated using ratings of the pain intensity, affective valence, arousal, and dominance of the painful and non-painful stimuli.

We hypothesized that the painful and non-painful stimuli in the EPSS would evoke the intended emotional feeling in participants. We tested whether different genders experienced different levels of perception and emotion in the EPSS. We intend to provide a well-validated and freely accessible stimuli database for research into the empathy for pain.

## Empathy for Limb Pain Picture Database (EPSS-Limb)

### Methods

#### Development of stimuli

##### Actors

Four actors (two females) at universities in Chongqing, China, aged 24–26 (mean = 25.34, standard deviation (SD) = 1.34), participated in the picture photograph sessions. All actors signed informed consent forms for the photo process in accordance with the Declaration of Helsinki.

##### Acting

**Fig. 1:**
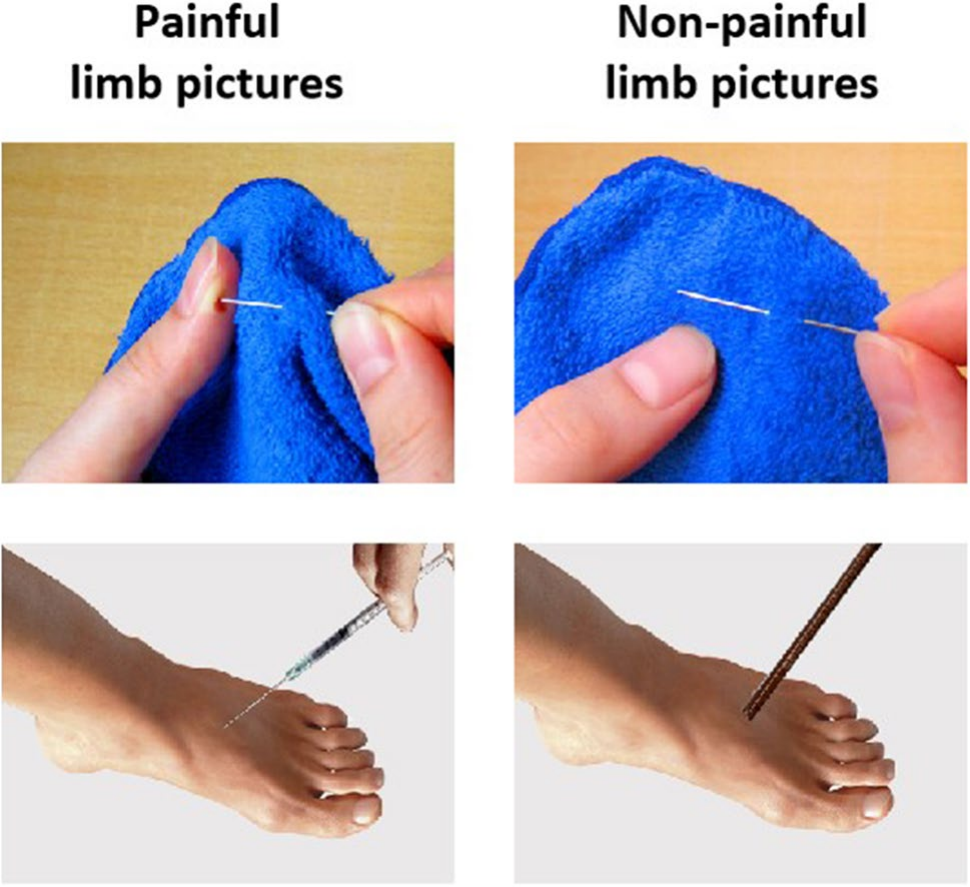
Examples of painful (*left panel*) and non-painful (*right panel*) limb pictures in the EPSS-Limb

In the photograph session, similar to the pictures used in our previously published studies (Meng et al., 2012; Meng et al., 2013; Meng et al., 2019b), the actors were instructed to produce painful and non-painful actions with their limbs (hands, feet, and forearms). All the pictures depicted familiar events that may happen in everyday life. Examples of painful limb pictures (see the left panel of Fig. 1) included a hand pricked by a needle and a foot penetrated by a syringe. The non-painful limb pictures corresponded to the painful limb pictures but without any nociceptive component. Examples of non-painful limb pictures included a hand using a needle to sew clothes and a foot touched by a pencil (see the right panel of Fig. 1). In the session for stimuli development, 213 original pictures exhibiting people’s limbs in painful and non-painful situations were taken.

##### Photographing and editing

A Sony camera (Sony Group Corporation) was used to take painful and non-painful limb pictures at a distance of approximately 1 m. Adobe Photoshop CS6 software (Adobe Systems Incorporated, San Jose, CA, USA) was used to edit the images. The luminance, contrast, and color of the painful and non-painful limb pictures were matched. Each picture was 9 × 6.76 cm (width × height) and 100 pixels per inch.

#### Stimulus validation

##### Participants

The validation procedure included 70 paid volunteers (35 women) from Chongqing Normal University, Chongqing, China. They were 18–27 years of age (mean = 23.01, SD = 1.95) and right-handed, with normal or corrected-to-normal vision. None of them had previously been diagnosed with a psychiatric, medical, or neurological disorder, nor did they have any painful symptoms from severe somatic diseases. Before validation, all the participants received a description of the stimulus validation procedure and signed an informed consent form. All the procedures were in accordance with the Declaration of Helsinki and approved by the Research Ethics Committee of Chongqing Normal University. The procedures were performed following ethical guidelines and regulations.

##### Procedure

The participants were seated in a quiet room with an ambient temperature of about 26 °C. The procedure was conducted using the E-Prime (3.0) program (Psychology Software Tools, Pittsburgh, PA, USA). Prior to the formal procedure, each participant participated in a training session to become familiar with the process (details see Appendix).

Each participant was instructed to evaluate the stimuli using four dimensions of rating scales: ① Pain intensity: Judge the perceived pain intensities of the models in the stimuli (1 = *no sensation,* 4 = *pain threshold*, 9 = *most intense pain imaginable*); ② Affective valence: Judge the perceived pleasure of the models in the stimuli (1 = *very unhappy*, 9 = *very happy*); ③ Arousal: Judge the perceived level of arousal of the models in the stimuli (1 = *extremely peaceful*, 9 = *extremely excited*); ④ Dominance: Judge the perceived level of control (1 = *extremely out of control*, 9 = *extremely in control*).

**Fig. 2:**
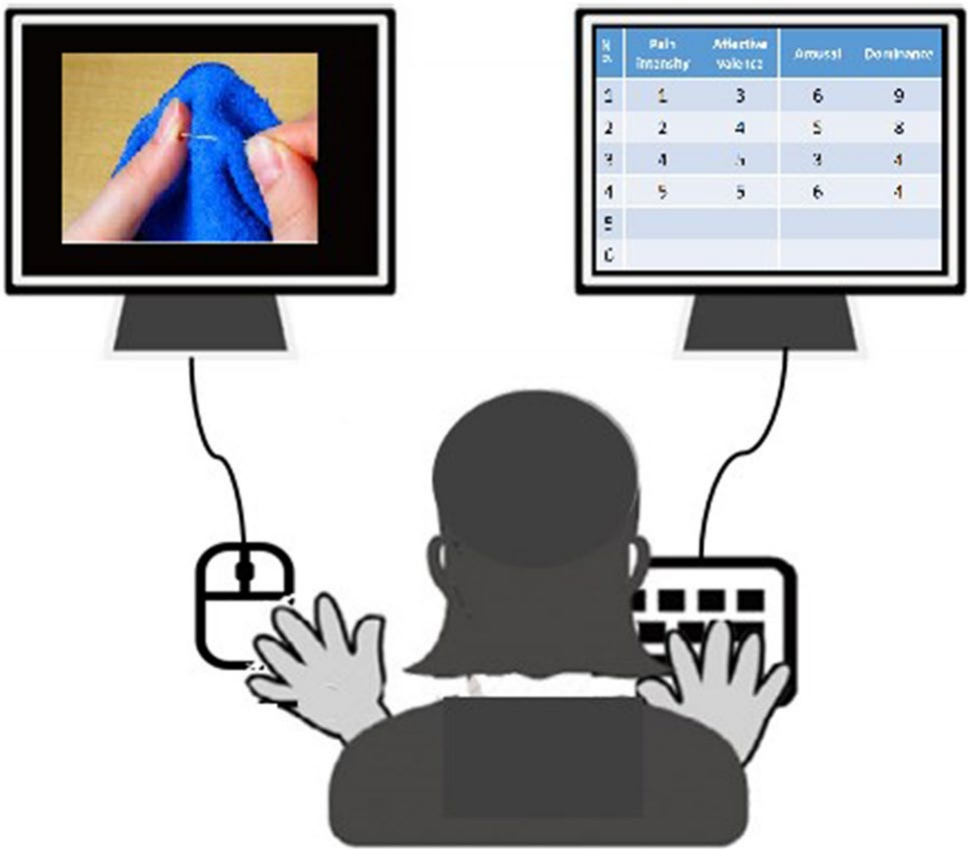
Procedure settings of stimuli validation of EPSS-Limb

As illustrated in Fig. 2, the participants were shown the stimuli on a computer screen. Then, a rating board with four dimensions of rating scales was displayed on the other computer screen. The participants were instructed to respond as accurately as possible to the stimuli by pressing a specific key (1 to 9) corresponding to the four dimensions of the rating scales. The order of the four dimensions of the rating scales was counterbalanced across all the participants to control for the order’s possible effects. The participants rated the stimuli on one dimension of the rating scale and moved to the next dimension. After completing the rating of one stimulus, the participants were instructed to click the mouse to rate the next stimulus. The stimuli were presented in a pseudorandomized order in 4 to 8 blocks. Each block lasted for < 10 min. The participants could take breaks at will between blocks to reduce fatigue and help them maintain attention on the rating scales. The rating was completed for each participant with several sessions of 1 to 2-h duration, spanning 1–3 days within a week.

##### Selection

The stimuli in the EPSS-Limb were selected using two criteria. First, the mean pain intensity rating scores for the painful stimuli had to be > 4 points, and the scores for the non-painful stimuli had to be < 4 points (4 is the pain threshold based on the rating scales of pain intensity). Second, rating scores ±3 SD away from the average for dimensions were excluded.

Based on a previous study (Yao et al., 2017), two different criteria were used to exclude participants. First, participants using the same response (e.g., 1) for more than 85% of the total responses for each dimension were excluded. Second, participants’ scores that were ±2.5 SD away from the dimension average were excluded.

##### Statistical analysis

The statistical analysis comprised four parts. First, the characteristics of the sub-database (i.e., the descriptive statistics of the painful and non-painful stimuli made by total, female, and male participants) of the four dimensions (pain intensity, affective valence, arousal, and dominance) were examined. Second, to ensure the reliability of the measures, the internal consistency of participant assessments was estimated by calculating split-half reliability scores with Spearman–Brown formula reliability scores (the participants were split into two subgroups of equal size according to a random procedure). Third, to explore the relationships between the dimensions, Pearson’s correlation coefficient was calculated, and interactive scatterplots were provided, where researchers could check the space location of each stimulus by considering the relationships of the four dimensions. Finally, to test dimensional ratings for painful and non-painful stimuli concerning the genders of participants and actors, the potential effect of stimuli type (painful vs. non-painful) × participant gender (female vs. male) × actor gender (female vs. male) on the participants’ assessments were calculated using repeated-measures analyses of variance (ANOVAs) in SPSS Statistics (IBM Corporation, Armonk, NY, USA).

### Results

Based on the selection criteria of the stimuli, 136 pictures (63.9% of the total stimuli) were selected for the EPSS-Limb, including 68 painful and 68 non-painful limb pictures. Based on the selection criteria of the participants, eight participants (11.4% of the total, four women) were excluded because of responses that formed a pattern with almost no variation or ±2.5 SD away from their group’s average. Thus, rating scores from 62 participants (31 women) aged 19–27 (mean = 23.24, SD = 1.89) were calculated.

#### Characteristics of the EPSS-Limb

The EPSS-Limb provides the number (N), mean, and SD of the rating scores for the painful and non-painful limb pictures across the four dimensions made by the total, female, and male participants (Table 1).

**Table 1:** Descriptive statistics for the EPSS-Limb (Mean ± SD)

Dimension	Stimulus type	*N*	Total	Female	Male
Pain intensity	Painful	68	5.77 ± 0.72	5.86 ± 0.76	5.68 ± 0.72
	Non-painful	68	1.79 ± 0.47	1.74 ± 0.52	1.85 ± 0.46
Affective valence	Painful	68	3.38 ± 0.41	3.89 ± 0.89	3.18 ± 0.47
	Non-painful	68	4.48 ± 0.46	4.26 ± 0.62	4.79 ± 0.61
Arousal	Painful	68	5.52 ± 0.58	5.46 ± 0.59	5.58 ± 0.60
	Non-painful	68	2.38 ± 0.47	2.31 ± 0.57	2.45 ± 0.45
Dominance	Painful	68	4.85 ± 0.61	4.74 ± 0.71	4.97 ± 0.55
	Non-painful	68	7.70 ± 0.40	7.78 ± 0.44	7.63 ± 0.43

#### Reliability of the measures of the EPSS-Limb

The internal consistency of participant assessments of the EPSS-Limb was estimated by calculating split-half reliability scores. The Spearman–Brown formula reliability scores were particularly high in four-dimensional ratings (pain intensity: *r* = 0.96; affective valence: *r* = 0.96; arousal: *r* = 0.99; dominance: *r* = 0.99). Therefore, the dimensional rating scores of participant assessments might be considered highly homogeneous in the EPSS-Limb.

#### Relationships among dimensions of the EPSS-Limb

**Fig. 3:**
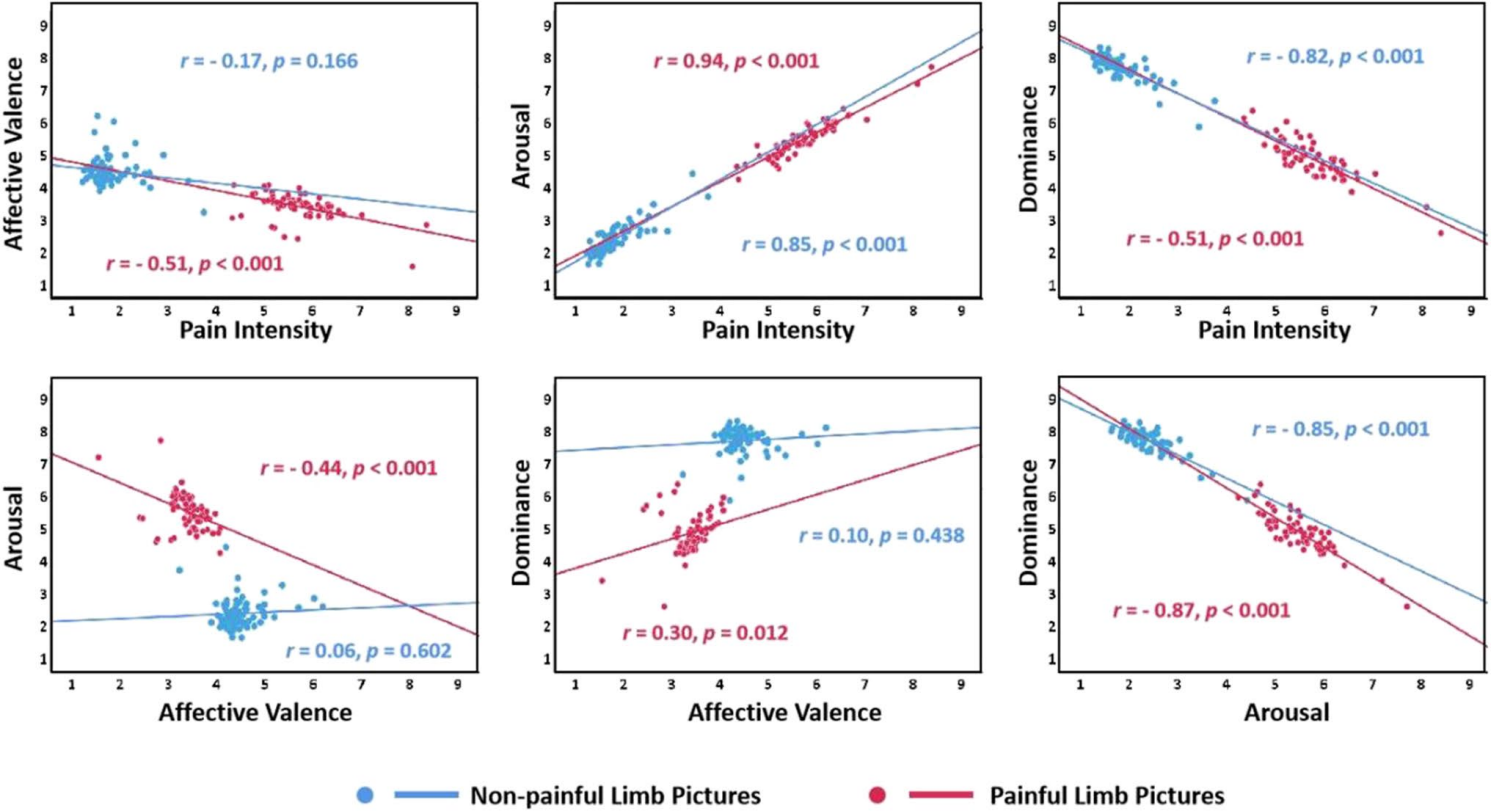
Scatterplot representing the distribution of the mean ratings in four dimensions provided in each painful (*red*) and non-painful (*blue*) limb picture of the EPSS-Limb

Figure 3 shows the relationships between the four dimensions of mean rating scores of painful and non-painful limb pictures for the EPSS-Limb. For the painful limb pictures, significant negative correlations were found in pain intensity × affective valence, pain intensity × dominance, arousal × affective valence, and arousal × dominance. Conversely, significant positive correlations were found in pain intensity × arousal and dominance × affective valence (all *p*s < 0.05). Concerning the non-painful limb pictures, significant negative correlations were found in pain intensity × dominance and arousal × dominance. Conversely, significant positive correlations were found in dominance × affective valence (all *p*s < 0.05).

#### Statistical analysis of the EPSS-Limb

Table 2 shows the summary of the statistical analysis of stimulus type × participant gender × actor gender in the participant assessments for four dimensions of the EPSS-Limb.

**Table 2:** Summary of the effect of stimulus type × participant gender × actor gender of the EPSS-Limb

	Pain intensity			Affective valence			Arousal			Dominance		
	*F*	*p*	η_p_^2^	*F*	*p*	η_p_^2^	*F*	*p*	η_p_^2^	*F*	*p*	η_p_^2^
Stimulus type	544.73	**< 0.001**	0.90	20.75	**< 0.001**	0.26	205.25	**< 0.001**	0.77	114.18	**< 0.001**	0.66
Participant gender	0.07	0.794	< 0.01	0.71	0.403	0.12	0.18	0.677	< 0.01	0.02	0.998	< 0.01
Actor gender	50.30	**< 0.001**	0.46	0.50	0.481	0.01	54.94	**< 0.001**	0.48	22.88	**< 0.001**	0.28
Stimulus type × Participant gender	0.71	0.403	0.01	12.70	**0.001**	0.18	< 0.01	0.980	< 0.01	0.52	0.474	0.01
Stimulus type × Actor gender	9.35	**0.003**	0.14	1.42	0.24	0.02	2.23	0.140	0.04	0.30	0.584	0.01
Participant gender × Actor gender	8.82	**0.004**	0.13	11.83	**0.001**	0.17	6.98	**0.010**	0.10	7.82	**0.007**	0.12
Stimulus type × Participant gender × Actor gender	1.19	0.281	0.02	11.91	**0.001**	0.17	0.78	0.382	0.01	0.06	0.808	< 0.01

The results were obtained using repeated-measures ANOVAs with stimulus type (painful vs. non-painful) × participant gender (female vs. male) × actor gender (female vs. male). Significant (*p* < 0.05) differences are indicated in boldface

##### Pain intensity

Pain intensity rating scores were modulated by stimulus type [*F*_(1,60)_ = 544.73, *p* < 0.001, η_p_^2^ = 0.90] and actor gender [*F*_(1,60)_ = 50.30, *p* < 0.001, η_p_^2^ = 0.46]. Painful limb pictures (5.78 ± 0.13) were more painful than non-painful limb pictures (1.81 ± 0.12). Pictures with male actors (3.91 ± 0.09) were deemed more painful than those of female actors (3.68 ± 0.09). Pain intensity rating scores were significantly influenced by the interaction of stimulus type × actor gender [*F*_(1,60)_ = 9.35, *p* = 0.003, η_p_^2^ = 0.14]. The differences between male and female actors tended to be smaller for painful limb pictures (male actor: 5.85 ± 0.12, female actor: 5.71 ± 0.13) than non-painful limb pictures (male actor: 1.97 ± 0.13, female actor: 1.65 ± 0.11). Pain intensity ratings were also influenced by the interaction of participant gender × actor gender [*F*_(1,60)_ = 8.82, *p* = 0.004, η_p_^2^ = 0.13]. The differences in rating scores between male and female actors made by female participants were larger than male participants (male participants: 0.13 ± 0.19, female participants: 0.32 ± 0.30; *p* = 0.004).

##### Affective valence

Affective valence rating scores were moderated by the main effect of stimulus type [*F*_(1,60)_ = 20.75, *p* < 0.001, η_p_^2^ = 0.26]. Painful limb pictures (3.39 ± 0.18) induced more negative feelings than non-painful limb pictures (4.48 ± 0.14). The interaction of stimulus type × participant gender [*F*_(1,60)_ = 12.70, *p* = 0.001, η_p_^2^ = 0.18] indicated that female participants rated the non-painful limb pictures more negatively than male participants (male participants: 5.00 ± 0.20, female participants: 3.97 ± 0.20; *p* < 0.001), while no difference was found for the painful limb pictures (male participants: 3.05 ± 0.25, female participants: 3.73 ± 0.25; *p* = 0.062). The interaction of participant gender × actor gender [*F*_(1,60)_ = 11.83, *p* = 0.001, η_p_^2^ = 0.17] indicated that for female participants, the rating scores for female actors were more negative than male actors (male actor: 3.93 ± 0.16, female actor: 3.76 ± 0.15; *p* = 0.005), while no difference was found for male participants (male actor: 3.97 ± 0.16, female actor: 4.08 ± 0.15; *p* = 0.058). In addition, the interaction of stimulus type × actor gender × participant gender [*F*_(1,60)_ = 11.91, *p* = 0.001, η_p_^2^ = 0.17] revealed that for non-painful limb pictures, male participants rated male actors more negatively than female actors (male actors: 4.85 ± 0.18, female actors: 5.15 ± 0.22; *p* = 0.003), while female participants rated female actors more negatively than male actors (male actors: 4.10 ± 0.18, female actors: 3.84 ± 0.22; *p* = 0.009). However, no such difference was found for the painful limb pictures (all *p*s > 0.05).

##### Arousal

Arousal rating scores were moderated by the main effects of stimulus type [*F*_(1,60)_ = 205.25, *p* < 0.001, η_p_^2^ = 0.77] and actor gender [*F*_(1,60)_ = 54.94, *p* < 0.001, η_p_^2^ = 0.48]. Painful limb pictures (5.53 ± 0.20) were more exciting than non-painful limb pictures (2.39 ± 0.16). Pictures with male actors (4.09 ± 0.15) were more exciting than female actors (3.84 ± 0.14). Arousal was also modulated by the interaction of actor gender × participant gender [*F*_(1,60)_ = 6.98, *p* = 0.010, η_p_^2^ = 0.10]. The differences in rating scores between male and female actors made by female participants were larger than male participants (male participants: 0.16 ± 0.23, female participants: 0.34 ± 0.30; *p* = 0.011).

##### Dominance

Dominance rating scores were moderated by the main effects of stimulus type [*F*_(1,60)_ = 114.18, *p* < 0.001, η_p_^2^ = 0.66] and actor gender [*F*_(1,60)_ = 22.88, *p* < 0.001, η_p_^2^ = 0.28]. Painful limb pictures (4.85 ± 0.25) were rated as more out of control than non-painful stimuli (7.69 ± 0.20). Pictures with male actors (6.17 ± 0.18) were rated as more out of control than female actors (6.36 ± 0.18). Dominance was also influenced by the interaction of actor gender × participant gender [*F*_(1,60)_ = 7.82, *p* = 0.007, η_p_^2^ = 0.16]. Female participants rated pictures with female actors as more in-control than male actors (male actors: 6.09 ± 0.26, female actors: 6.39 ± 0.26; *p* < 0.001), while no difference was found for male participants (male actor: 6.26 ± 0.26, female actor: 6.33 ± 0.26; *p* = 0.165).

## Empathy for Face Pain Picture Database (EPSS-Face)

### Method

#### Development of stimuli

**Fig. 4:**
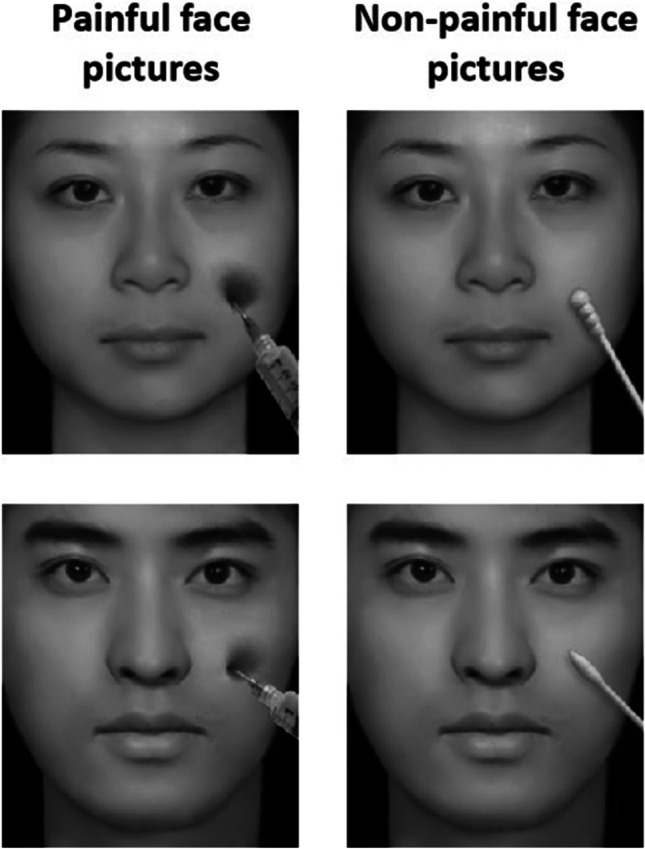
Examples of painful (*left panel*) and non-painful (*right panel*) face pictures in the EPSS-Face

The EPSS-Face database contains 160 digital pictures of faces in painful or non-painful situations. Some of the stimuli have been used in previously published studies (Li et al., 2020; Meng et al., 2020a; Yang et al., 2022). These stimuli were revised from a picture database that had been previously validated, in which the images of the faces were morphed pictures with given black backgrounds and shown in grayscale (Hu et al., 2018; Yang et al., 2015). The EPSS-Face comprises pictures of 80 painful faces (40 female and 40 male) and 80 non-painful faces (40 female and 40 male). The painful face pictures depict pain by penetrating the model’s cheek with a needle (see the left panel of Fig. 4), and the non-painful face pictures show the model’s face being touched with a Q-tip (see the right panel of Fig. 4). The pictures were edited using Adobe Photoshop CS6. The luminance, contrast, and color of the painful and non-painful pictures were matched. Each picture was 6.88 cm × 7.94 cm (width × height), with 96 pixels per inch.

#### Stimulus validation

##### Participants

The validation procedure included 70 paid volunteers (35 women) from Chongqing Normal University, Chongqing, China. They were aged 18–30 (mean = 22.29, SD = 2.53) and right-handed, with normal or corrected-to-normal vision. The other inclusion criteria were similar to those for the EPSS-Limb.

##### Procedures, selection criteria, and statistical analysis

The evaluation procedure, selection criteria of stimuli and participants, and statistical analysis methods for the EPSS-Face were similar to those for the EPSS-Limb.

### Results

Based on the selection criteria, all the pictures were selected for the EPSS-Face, including 80 painful and 80 non-painful face pictures. In addition, the participants’ rating data were included for further statistical analyses. Examples of painful and non-painful face pictures are shown in Fig. 4.

#### Characteristics of the EPSS-Face

The EPSS-Face provides the descriptive statistics results of the rating scores for the painful and non-painful face pictures across the four dimensions made by the total, female, and male participants (Table 3).

**Table 3:** Descriptive statistics for the EPSS-Face (Mean ± SD)

Dimension	Stimulus type	*N*	Total	Female	Male
Pain intensity	Painful	80	6.05 ± 0.33	6.02 ± 0.40	6.07 ± 0.35
	Non-painful	80	1.39 ± 0.12	1.36 ± 0.15	1.41 ± 0.15
Affective valence	Painful	80	3.33 ± 0.23	3.30 ± 0.30	3.36 ± 0.22
	Non-painful	80	4.94 ± 0.25	5.00 ± 0.34	4.87 ± 0.24
Arousal	Painful	80	4.98 ± 0.22	4.75 ± 0.29	5.22 ± 0.22
	Non-painful	80	2.65 ± 0.15	2.72 ± 0.22	2.58 ± 0.16
Dominance	Painful	80	5.02 ± 0.23	4.96 ± 0.31	5.09 ± 0.24
	Non-painful	80	7.42 ± 0.15	7.14 ± 0.23	7.70 ± 0.17

#### Reliability of the measures of the EPSS-Face

The internal consistency of participant assessments of the EPSS-Face was estimated by calculating split-half reliability scores. The Spearman–Brown formula reliability scores were particularly high in four-dimensional ratings (pain intensity: *r* = 0.98; affective valence: *r* = 0.97; arousal: *r* = 0.99; dominance: *r* = 0.99). Therefore, dimensional ratings of participant assessments might be considered highly homogeneous in the EPSS-Face.

#### Relationships between the dimensions of the EPSS-Face

**Fig. 5:**
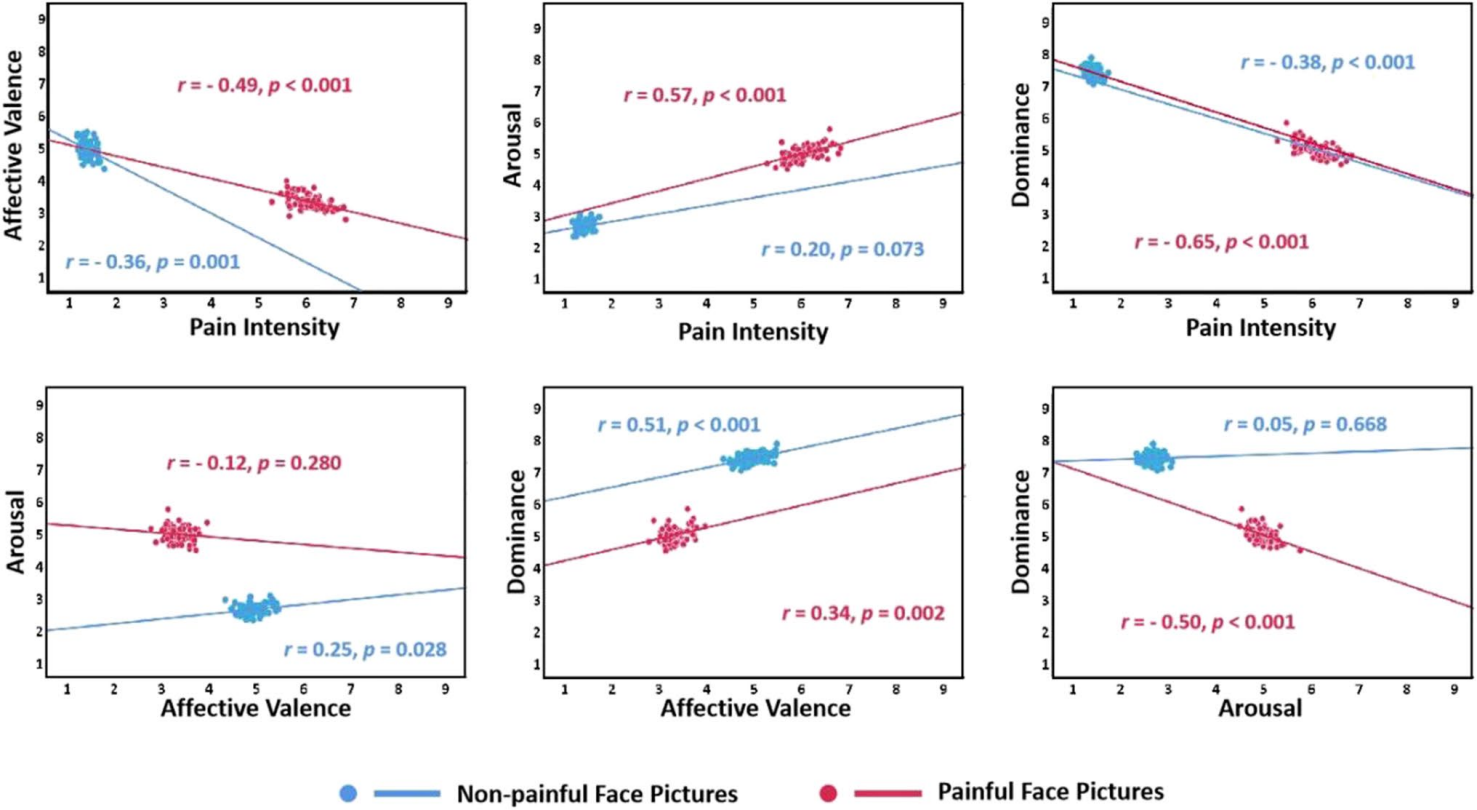
Scatterplot representing the distribution of the mean rating in four dimensions provided in each painful (*red*) and non-painful (*blue*) face picture of the EPSS-Face

Figure 5 shows the relationships between the four dimensions of mean rating scores of painful and non-painful face pictures for the EPSS-Face. Concerning the painful face pictures, significant negative correlations were found in pain intensity × affective valence, pain intensity × dominance, and arousal × dominance. In contrast, significant positive correlations were found in pain intensity × arousal and dominance × affective valence (all *p*s < 0.05). For the non-painful face pictures, significant negative correlations were found in pain intensity × affective valence, and pain intensity × dominance. In addition, significant positive correlations were found in affective valence × arousal and dominance × affective valence (all *p*s < 0.05).

#### Statistical analysis of the EPSS-Face

Table 4 shows the summary of the statistical analysis of stimulus type × participant gender × actor gender in the participant assessments for four dimensions of the EPSS-Face.

**Table 4:** Summary of the effect of stimulus type × participant gender × actor gender of the EPSS-Face

	Pain intensity			Affective valence			Arousal			Dominance		
	*F*	*p*	η_p_^2^	*F*	*p*	η_p_^2^	*F*	*p*	η_p_^2^	*F*	*p*	η_p_^2^
Stimulus type	921.30	**< 0.001**	0.93	81.96	**< 0.001**	0.55	72.40	**< 0.001**	0.52	67.74	**< 0.001**	0.50
Participant gender	0.09	0.768	< 0.01	0.06	0.807	< 0.01	0.32	0.576	0.01	1.18	0.282	0.02
Actor gender	6.58	**0.013**	0.09	7.57	**0.008**	0.10	0.25	0.618	< 0.01	1.82	0.182	0.03
Stimulus type × Participant gender	< 0.01	0.955	< 0.01	0.30	0.586	< 0.01	1.19	0.280	0.02	0.52	0.473	0.01
Stimulus type × Actor gender	0.03	0.869	< 0.01	4.89	**0.030**	0.07	3.69	0.059	0.05	4.67	**0.034**	0.06
Participant gender × Actor gender	8.66	**0.004**	0.11	5.14	**0.027**	0.07	0.18	0.670	< 0.01	1.31	0.256	0.02
Stimulus type × Participant gender × Actor gender	5.01	**0.028**	0.07	0.57	0.452	0.01	2.63	0.110	0.04	3.66	0.060	0.05

The results were obtained using repeated-measures ANOVAs with stimulus type (painful vs. non-painful) × participant gender (female vs. male) × actor gender (female vs. male). Significant (*p* < 0.05) differences are indicated in boldface

##### Pain intensity

Pain intensity rating scores were moderated by the main effects of stimulus type [*F*_(1,68)_ = 921.30, *p* < 0.001, η_p_^2^ = 0.93] and actor gender [*F*_(1,68)_ = 6.58, *p* =0.013, η_p_^2^ = 0.09]. Painful face pictures (6.04 ± 0.14) were more painful than the non-painful face pictures (1.38 ± 0.07). Pictures with male actors (3.74 ± 0.08) were deemed more painful than female actors (3.69 ± 0.08). The interaction of actor gender × participant gender [*F*_(1,68)_ = 8.66, *p* =0.004, η_p_^2^ = 0.11] indicated that male participants rated male actors (3.79 ± 0.12) as more painful than female actors (3.68 ± 0.11), while no difference was found for female participants (male actor: 3.69 ± 0.12, female actor: 3.69 ± 0.11; *p* = 0.790). The interaction of stimulus type × actor gender × participant gender [*F*_(1,68)_ = 5.01, *p* = 0.028, η_p_^2^ = 0.07] was significant. For non-painful face pictures, male participants rated male actors as being more painful than female actors (male actors: 1.45 ± 0.10, female actors: 1.38 ± 0.09; *p* = 0.025), while no difference was found in female participants (male actors: 1.38 ± 0.10, female actors: 1.33 ± 0.09; *p* = 0.153). For painful face pictures, male participants rated male actors as being more painful than female actors (male actors: 6.14 ± 0.21, female actors: 5.99 ± 0.20; *p* = 0.003), while no such difference was found in female participants (male actors: 6.00 ± 0.21, 6.06 ± 0.20; *p* = 0.252). In addition, the differences in rating scores between painful and non-painful face pictures with male actors were larger than female actors for female participants (male actors: 4.70 ± 1.44, female actors: 4.61 ± 1.30; *p* = 0.037), while no such difference was found in male participants (male actors: 4.62 ± 1.24, female actors: 4.72 ± 1.19; *p* = 0.222).

##### Affective valence

Affective valence rating scores were moderated by the main effects of stimulus type [*F*_(1,68)_ = 81.96, *p* < 0.001, η_p_^2^ = 0.55] and actor gender [*F*_(1,68)_ = 7.57, *p* = 0.008, η_p_^2^ = 0.10]. Painful face pictures (3.33 ± 0.16) were rated as more negative than non-painful face pictures (4.94 ± 0.08). Pictures with male actors (4.09 ± 0.09) were rated as more negative than female actors (4.18 ± 0.10). The interaction of actor gender × participant gender [*F*_(1,68)_ = 5.14, *p* = 0.027, η_p_^2^ = 0.07] indicated that female participants rated male actors as more negative than female actors (male actors: 4.08 ± 0.12, female actors: 4.23 ± 0.14; *p* = 0.001), while no difference was found in the male participants (male actors: 4.11 ± 0.12, female actors: 4.12 ± 0.14; p = 0.733). The interaction of stimuli type × actor gender [*F*_(1,68)_ = 4.89, *p* = 0.030, η_p_^2^ = 0.07] indicated that for the non-painful face pictures, pictures with female actors were rated as less negative than male actors (male actors: 4.88 ± 0.08, female actors: 5.01 ± 0.09; *p* = 0.001), while no difference was found for the painful face pictures (male actors: 3.31 ± 0.15, female actors: 3.35 ± 0.17; *p* = 0.285).

##### Arousal

Arousal rating scores were moderated by the main effect of stimulus type [*F*_(1,68)_ = 72.40, *p* < 0.001, η_p_^2^ = 0.52]. Painful face pictures (4.99 ± 0.23) were rated as more exciting than non-painful face pictures (2.65 ± 0.18).

##### Dominance

Dominance rating scores were moderated by the main effect of stimulus type [*F*_(1,68)_ = 67.34, *p* < 0.001, η_p_^2^ = 0.50], and painful face pictures (5.02 ± 0.25) were rated as less in control than non-painful face pictures (7.43 ± 0.18). The interaction of stimulus type × actor gender [*F*_(1,68)_ = 4.67, *p* = 0.034, η_p_^2^ = 0.06] indicated that for the non-painful face pictures, pictures with female actors were rated as more in control than male actors (male actors: 7.38 ± 0.18, female actors: 7.47 ± 0.18; *p* = 0.017), while no difference was found for the painful face pictures (male actors: 5.03 ± 0.24, female actors: 5.02 ± 0.25; *p* = 0.808).

## Empathy for Voice Pain Database (EPSS-Voice)

### Method

#### Development of stimuli

##### Actors

Thirty-one actors (15 females) studying drama at universities in Chongqing, China, aged 18–23 (mean = 21.13, SD = 1.41), participated in the recording sessions. All the actors had normal vision and hearing and signed informed consent forms to participate in the recording process in accordance with the Declaration of Helsinki. They received ￥100 in compensation.

##### Acting

The actors were instructed to produce short vocal exclamations that were either painful or neutral using the vowel sound /a/. A short rehearsal session preceded each recording round, during which the sounds’ level and duration were adjusted. Each vocalization category was performed several times until our qualitative criterion was reached. Two experimenters had to be able to recognize the painful or non-painful voices the actors produced. Feedback was given to the actors during the session so that they could improve their performance. Finally, 120 vocalizations (89 painful and 31 non-painful) were recorded and categorized as painful and non-painful voices, respectively.

##### Recording and editing

**Fig. 6:**
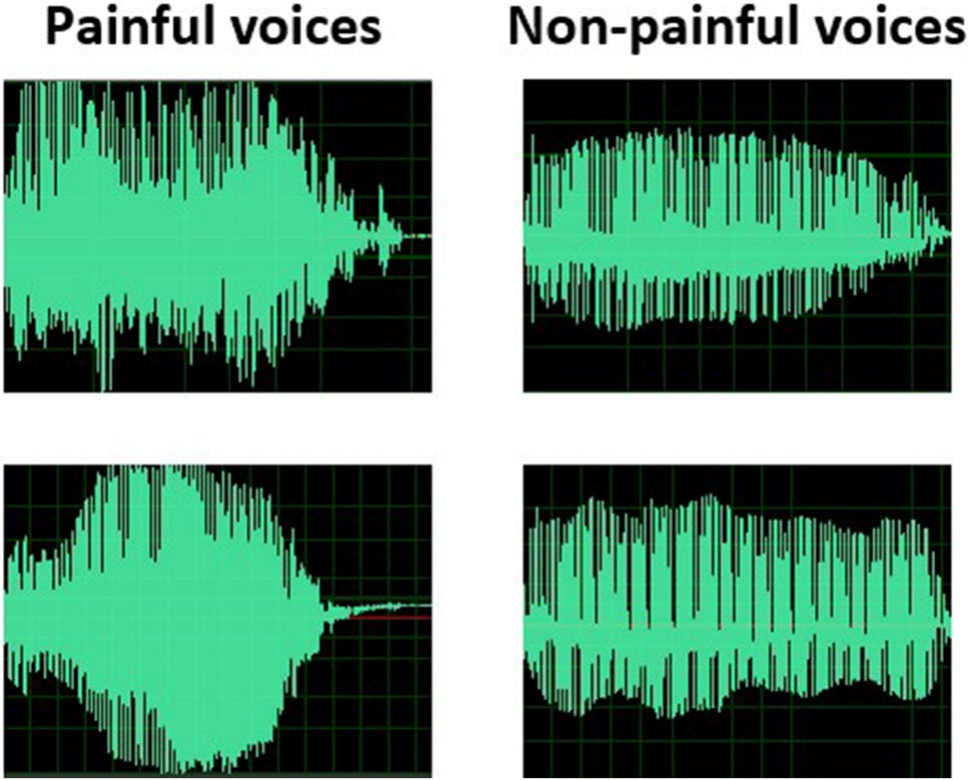
Examples of painful (*left panel*) and non-painful (*right panel*) voice waveforms in the EPSS-Voice

The voices were recorded in a sound-proof room using an ATR2500 condenser microphone (Audio Technica) at a distance of approximately 40 cm. The recordings were edited into short, meaningful segments of about 1 s each. Their peak values were normalized, and they were down-sampled at 44.1 kHz using Adobe Audition (Adobe Systems, Inc.). Only the best example for each actor and vocalization category was kept for the validation stage (Fig. 6).

#### Stimuli validation

##### Participants

Seventy adults (35 females) from Chongqing Normal University, Chongqing, China, were recruited as paid participants. They were aged 18–31 (mean = 22.89, SD = 2.32) and right-handed, with normal vision and hearing. The other inclusion criteria were the same as for the EPSS-Limb.

##### Procedures, selection criteria, and statistical analysis

**Fig. 7:**
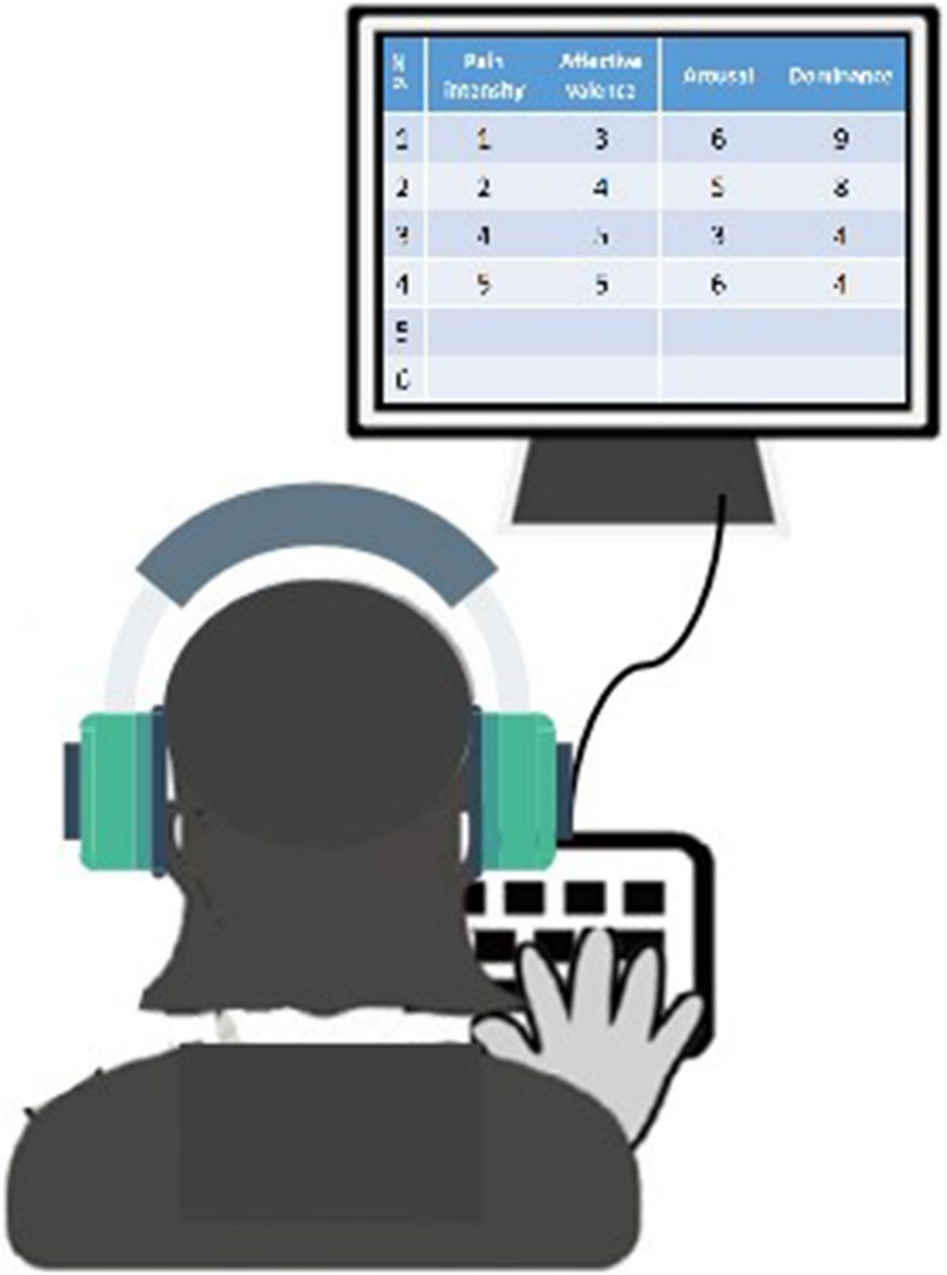
Procedure settings of stimuli validation of EPSS-Voice

The main evaluation procedure for the EPSS-Voice was similar to those for the EPSS-Limb, except that the painful and non-painful voices were presented through headphones (Fig. 7). In addition, the selection criteria and statistical analysis methods for the EPSS-Voice were similar to those for the EPSS-Limb.

### Results

Based on the selection criteria of the stimuli, 15 male and 15 female actors who produced the most successful displays were selected. Sixty voices were selected to form the EPSS-Voice, including 30 painful (see the left panel of Fig. 6) and 30 non-painful voices (see the right panel of Fig. 6). Based on the selection criteria of the participants, ten participants (14.3% of the total, five women) were excluded. Thus, rating scores from 60 participants (30 females) aged 19–31 (mean = 22.77, SD = 2.27) were calculated.

#### Characteristics of the EPSS-Voice

The EPSS-Voice provides the descriptive statistics results of the rating scores for the painful and non-painful voices across the four dimensions made by the total, female, and male participants (Table 5).

**Table 5:** Descriptive statistics for the EPSS-Voice (Mean ± SD)

Dimension	Stimuli type	*N*	Total	Female	Male
Pain intensity	Painful	30	5.80 ± 0.92	5.80 ± 1.01	5.79 ± 0.88
	Non-painful	30	1.60 ± 0.16	1.52 ± 0.18	1.69 ± 0.19
Affective valence	Painful	30	3.68 ± 0.69	3.57 ± 0.75	3.79 ± 0.68
	Non-painful	30	5.18 ± 0.13	5.23 ± 0.15	5.12 ± 0.15
Arousal	Painful	30	4.98 ± 0.49	4.91 ± 0.63	5.05 ± 0.49
	Non-painful	30	3.87 ± 0.19	4.04 ± 0.16	3.70 ± 0.29
Dominance	Painful	30	4.98 ± 0.88	4.81 ± 0.92	5.15 ± 0.88
	Non-painful	30	7.90 ± 0.17	8.00 ± 0.18	7.81 ± 0.27

#### Reliability of the measures of the EPSS-Voice

The internal consistency of participant assessments of the EPSS-Voice was estimated by calculating split-half reliability scores. The Spearman–Brown formula reliability scores were particularly high in four-dimensional ratings (pain intensity: *r* = 0.94; affective valence: *r* = 0.92; arousal: *r* = 0.95; dominance: *r* = 0.99). Therefore, dimensional ratings of participant assessments might be considered highly homogeneous in the EPSS-Voice.

#### Relationships between dimensions of the EPSS-Voice

**Fig. 8:**
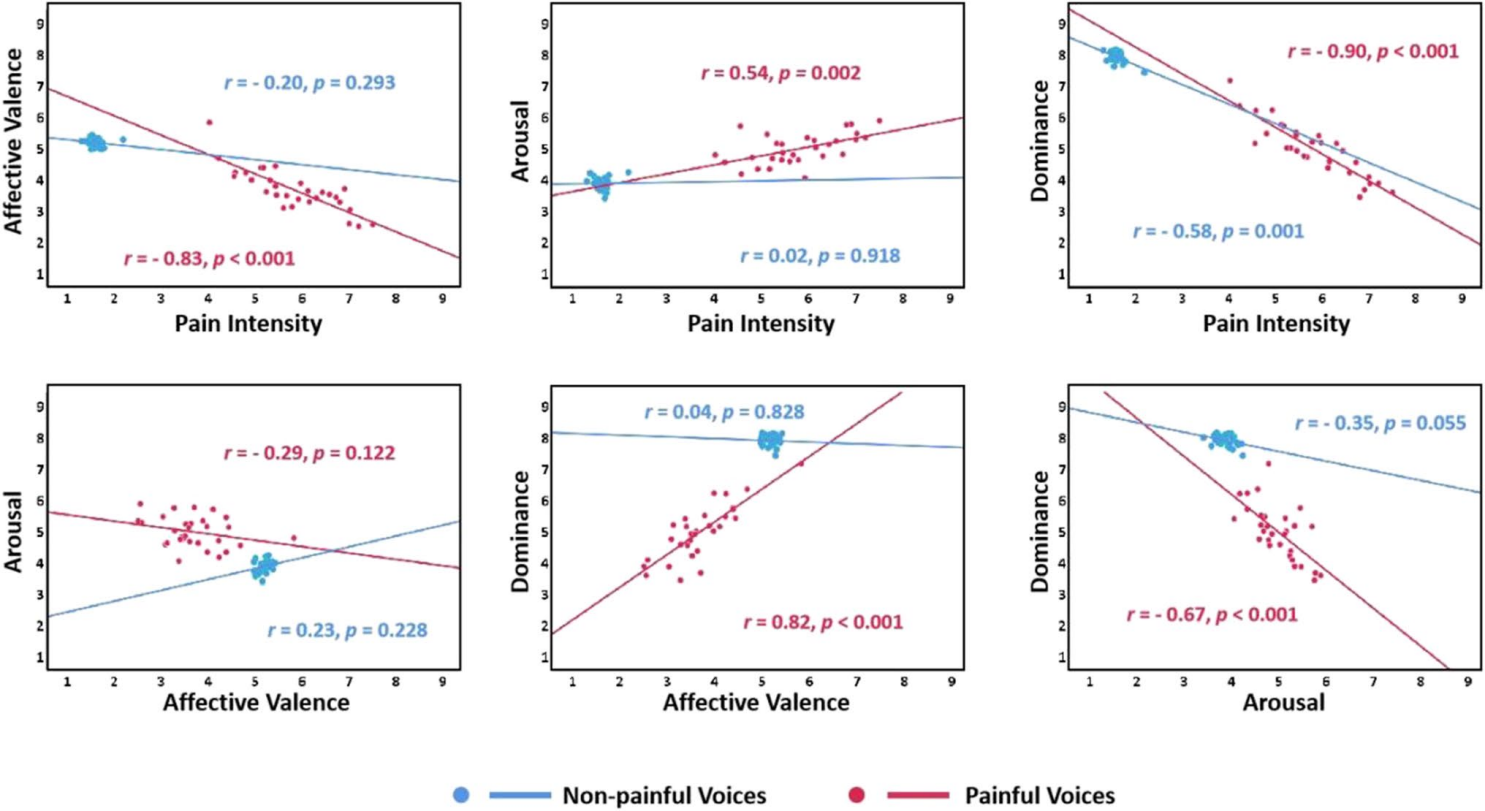
Scatterplot representing the distribution of the mean ratings in the four dimensions provided in each painful (*red*) and non-painful (*blue*) voice of the EPSS-Voice

Figure 8 shows the relationships between the four dimensions of mean rating scores of painful and non-painful voices for the EPSS-Voice. Concerning the painful voices, significant negative correlations were found in pain intensity × affective valence, pain intensity × dominance, and arousal × dominance. In contrast, significant positive correlations were found in pain intensity × arousal and dominance × affective valence (all *p*s < 0.05). Concerning the non-painful voices, a significant negative correlation was found in pain intensity × dominance (*p* < 0.05).

#### Statistical analysis of the EPSS-Voice

Table 6 shows the summary of the statistical analysis of stimulus type × participant gender × actor gender in the participant assessments for four dimensions of the EPSS-Voice.

**Table 6:** Summary of the effect of stimulus type × participant gender × actor gender in the EPSS-Voice

	Pain intensity			Affective valence			Arousal			Dominance		
	*F*	*p*	η_p_^2^	*F*	*p*	η_p_^2^	*F*	*p*	η_p_^2^	*F*	*p*	η_p_^2^
Stimulus type	1224.42	**< 0.001**	0.96	158.34	**< 0.001**	0.73	13.51	**0.001**	0.19	166.85	**< 0.001**	0.74
Participant gender	0.43	0.516	0.01	0.15	0.700	< 0.01	0.19	0.662	< 0.01	0.05	0.823	< 0.01
Actor gender	38.46	**< 0.001**	0.40	19.00	**< 0.001**	0.25	3.87	0.054	0.06	31.61	**< 0.001**	0.35
Stimulus type × Participant gender	0.53	0.470	0.01	1.95	0.168	0.03	0.64	0.427	0.01	1.39	0.243	0.02
Stimulus type × Actor gender	42.36	**< 0.001**	0.42	30.53	**< 0.001**	0.35	3.64	0.061	0.06	50.91	**< 0.001**	0.47
Participant gender × Actor gender	1.13	0.292	0.02	2.01	0.162	0.03	1.59	0.212	0.03	1.00	0.322	0.02
Stimulus type × Participant gender × Actor gender	0.27	0.604	0.01	1.37	0.247	0.02	0.45	0.506	0.01	0.01	0.925	< 0.01

The results were obtained using repeated-measures ANOVAs with stimulus type (painful vs. non-painful) × participant gender (female vs. male) × actor gender (female vs. male). Significant (*p* < 0.05) differences are indicated in boldface

##### Pain intensity

Pain intensity rating scores were moderated by the main effects of stimulus type [*F*_(1,58)_ = 1224.42, *p* < 0.001, η_p_^2^ = 0.96] and actor gender [*F*_(1,58)_ = 38.46, *p* < 0.001, η_p_^2^ = 0.40]. Painful voices (5.80 ± 0.08) were more painful than non-painful voices (1.60 ± 0.09). Voices with female actors (3.83 ± 0.07) were more painful than male actors (3.57 ± 0.06). The interaction of stimuli type × actor gender [*F*_(1,60)_ = 42.36, *p <* 0.001, η_p_^2^ = 0.42] indicated that for the painful voices, voices with female actors were more painful than male actors (male actors: 5.53 ± 0.08, female actors: 6.06 ± 0.10; *p* <0.001), while no difference was found for the non-painful voices (male actors: 1.61 ± 0.09, female actors: 1.59 ± 0.09; *p* = 0.766).

##### Affective valence

Affective valence rating scores were moderated by the main effects of stimulus type [*F*_(1,58)_ = 158.34, *p* < 0.001, η_p_^2^ = 0.73] and actor gender [*F*_(1,58)_ = 19.00, *p* < 0.001, η_p_^2^ = 0.25]. Painful voices (1.60 ± 0.09) were more negative than non-painful voices (5.80 ± 0.08). Voices with female actors (4.32 ± 0.08) were more negative than male actors (4.54 ± 0.07). The interaction of stimulus type × actor gender [*F*_(1,58)_ = 30.53, *p <* 0.001, η_p_^2^ = 0.35] indicated that concerning painful voices, voices with female actors were more negative than male actors (male actors: 3.92 ± 0.10, female actors: 3.44 ± 0.14, *p* < 0.001), while no difference was found in non-painful voices (male actors: 5.16 ± 0.07, female actors: 5.19 ± 0.07; *p* = 0.611).

##### Arousal

Arousal rating scores were moderated by the main effect of stimulus type [*F*_(1,58)_ = 13.51, *p* = 0.001, η_p_^2^ = 0.19]. Painful voices (4.98 ± 0.18) were more exciting than non-painful voices (3.87 ± 0.20).

##### Dominance

Dominance rating scores were significantly moderated by the main effects of stimulus type [*F*_(1,58)_ = 166.85, *p* < 0.001, η_p_^2^ = 0.74] and actor gender [*F*_(1,58)_ = 31.61, *p* < 0.001, η_p_^2^ = 0.35]. Painful voices (4.98 ± 0.19) were rated as more out of control than non-painful voices (7.90 ± 0.20). However, the voices of male actors (6.70 ± 0.17) were rated as more in control than female actors (6.19 ± 0.16). The interaction of stimulus type × actor gender [*F*_(1,58)_ = 50.91, *p <* 0.001, η_p_^2^ = 0.47] indicated that concerning painful voices, the voices of male actors were rated as more in control than female actors (male actors: 5.42 ± 0.20, female actors: 4.53 ± 0.20, *p* <0.001), while no difference was found for non-painful voices (male actors: 7.97 ± 0.20, female actors: 7.84 ± 0.20; *p* = 0.127).

## Empathy for Action Pain Video Database (EPSS-Action_Video)

### Method

#### Development of stimuli

##### Actors

Twenty actors studying drama at universities in Chongqing, China, aged 18–24 (mean = 22.30, SD = 2.12), participated in the video recording sessions. All the actors signed informed consent forms to participate in the recording process in accordance with the Declaration of Helsinki and the portrait agreement. They received ￥200 for their performance.

##### Acting

**Fig. 9:**
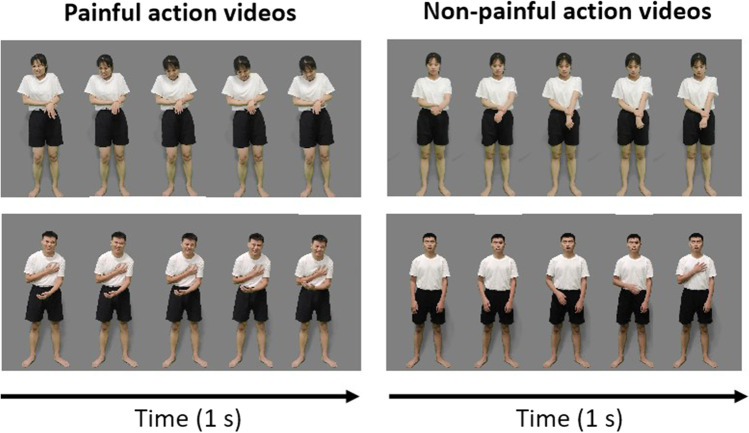
An illustration of sample frames from painful (*left panel*) and non-painful (*right panel*) action videos in the EPSS-Action_Video

The actors were instructed to produce painful and non-painful actions in the filming session. The actions used 12 body parts (i.e., head, teeth, neck, arms, elbows, hands, belly, knees, genitalia, chest, waist, and hip). In the session of stimuli development, 480 painful actions and 480 non-painful actions were filmed, the examples of which are illustrated in Fig. 9.

##### Filming and editing

Before filming, the actors were asked to dress in uniforms (a white T-shirt and black shorts). They were also asked to take off their accessories and not to wear make-up. The actors were filmed in an evenly lit green-screen studio with an ambient temperature of approximately 26°C. A Sony FDR-AXP55 (Sony Group Corporation) camera was used at a distance of approximately 3 m. Each film was 2160 × 1280 pixels, with a 60-fps progressive scan. The height of the camera was 1.2 m.

Adobe Premiere Pro2020 (Adobe Systems Incorporated) was used to edit the video footage. The green background was changed into a gray background, and the actor was isolated from all other contextual information. Each video was edited to a duration of 1 s. It was saved in mp4 format at 768 × 432 pixels and 60 fps.

#### Validation

##### Participants

Seventy adults (34 females) from Chongqing Normal University, Chongqing, China, were recruited as paid participants. They were aged 18–30 (mean = 23.23, SD = 2.15) and right-handed, with normal or corrected-to-normal vision. The other inclusion criteria were similar to the EPSS-Limb.

##### Procedures, selection criteria, and statistical analysis

The evaluation procedure, selection criteria of stimuli, participants, and statistical analysis methods for the EPSS-Action_Video were similar to those for the EPSS-Limb.

### Results

Based on the selection criteria of the stimuli, 239 painful and 239 non-painful action videos, recorded by 20 actors (ten females), were selected (illustrated in Fig. 9). Based on the selection criteria of the participants, ten participants (14.3% of the total, four women) were excluded. Thus, rating scores from 60 participants (30 females) aged 18–25 (mean = 21.80, SD = 1.90) were calculated.

#### Characteristics of the EPSS-Action_Video

The EPSS-Action_Video provides the descriptive statistics results of the rating scores for the painful and non-painful action videos across the four dimensions made by the total, female, and male participants (Table 7).

**Table 7:** Descriptive statistics for the EPSS-Action_Video (Mean ± SD)

Dimension	Stimulus type	*N*	Total	Female	Male
Pain intensity	Painful	239	6.31 ± 0.68	6.43 ± 0.65	6.19 ± 0.76
	Non-painful	239	2.24 ± 0.35	2.17 ± 0.39	2.31 ± 0.36
Affective valence	Painful	239	3.88 ± 0.25	3.68 ± 0.30	4.09 ± 0.32
	Non-painful	239	5.01 ± 0.17	4.96 ± 0.17	5.06 ± 0.28
Arousal	Painful	239	4.84 ± 0.43	4.72 ± 0.40	4.96 ± 0.50
	Non-painful	239	3.11 ± 0.22	3.11 ± 0.21	3.10 ± 0.32
Dominance	Painful	239	5.88 ± 0.32	6.07 ± 0.37	5.69 ± 0.36
	Non-painful	239	7.02 ± 0.18	7.23 ± 0.16	6.81 ± 0.28

#### Reliability of the measurements of the EPSS-Action_Video

The internal consistency of participant assessments of the EPSS-Action_Video was estimated by calculating split-half reliability scores. The Spearman–Brown formula reliability scores were particularly high in four-dimensional ratings (Pain intensity: *r* = 0.99; Affective valence: *r* = 0.99; Arousal: *r* = 0.99; Dominance: *r* = 0.99). Therefore, dimensional ratings of participant assessments might be considered highly homogeneous in the EPSS-Action_Video.

#### Relationships between the dimensions of the EPSS-Action_Video

**Fig. 10:**
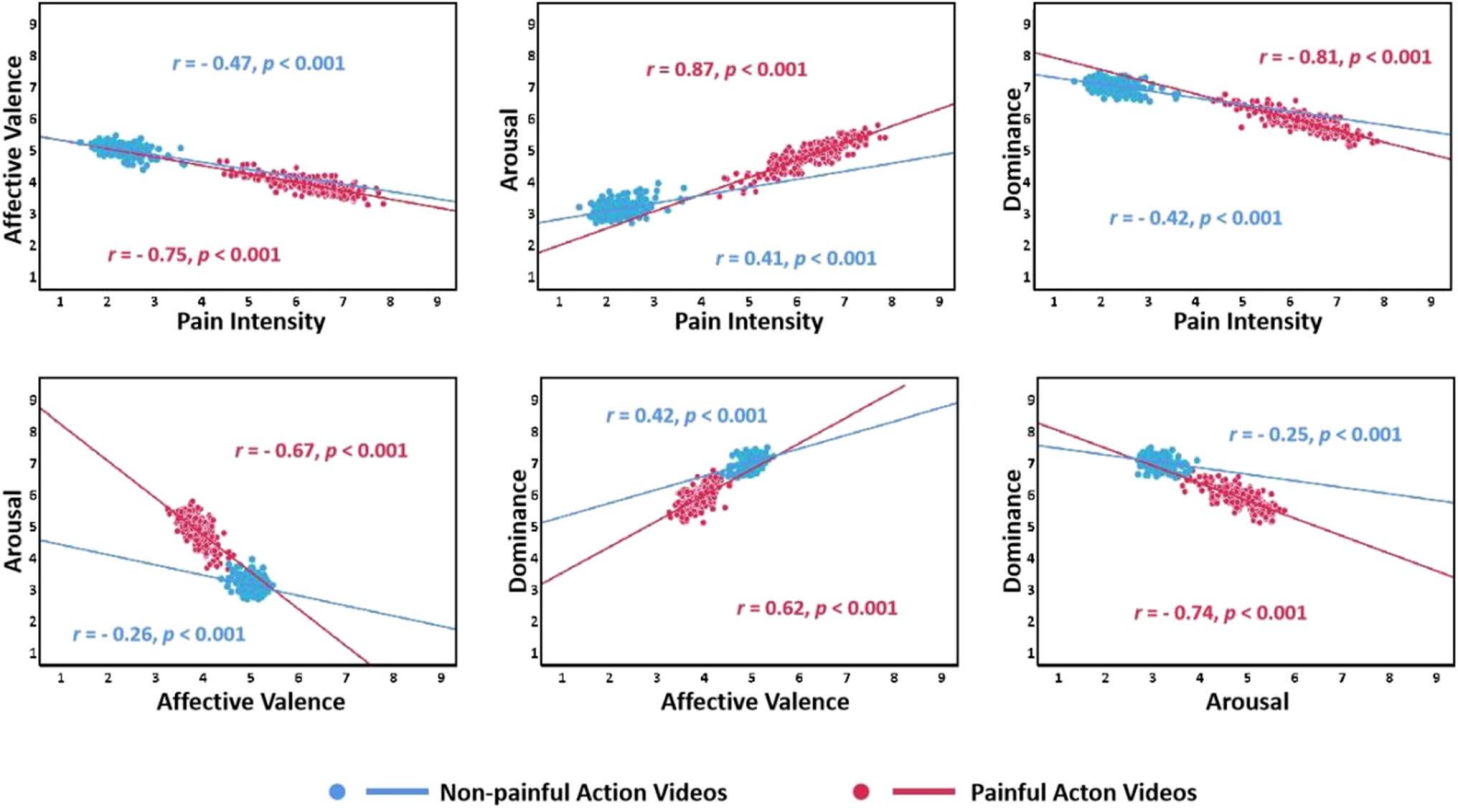
Scatterplot representing the distribution of the mean ratings in four dimensions provided in each painful (*red*) and non-painful (*blue*) action video of the EPSS-Action_Video

Figure 10 shows the relationships between the four dimensions of mean rating scores of painful and non-painful action videos for the EPSS-Action_Video. Concerning both the painful and non-painful action videos, significant negative correlations were found in pain intensity × affective valence, pain intensity × dominance, arousal × affective valence, and arousal × dominance. In addition, significant positive correlations were found in pain intensity × arousal and dominance × affective valence (all *p*s < 0.001).

#### Statistical analysis of the EPSS-Action_Video

Table 8 shows the summary of the statistical analysis of stimulus type × participant gender × actor gender in the participant assessments for four dimensions of the EPSS-Action_Video.

**Table 8:** Summary of the effect of stimulus type × participant gender × actor gender of the EPSS-Action_Video

	Pain intensity			Affective valence			Arousal			Dominance		
	*F*	*p*	η_p_^2^	*F*	*p*	η_p_^2^	*F*	*p*	η_p_^2^	*F*	*p*	η_p_^2^
Stimulus type	831.44	**< 0.001**	0.94	31.28	**< 0.001**	0.35	52.60	**< 0.001**	0.48	20.19	**< 0.001**	0.26
Participant gender	0.05	0.832	< 0.01	2.27	0.137	0.04	0.16	0.736	< 0.01	0.83	0.367	0.01
Actor gender	1.23	0.273	0.02	0.02	0.886	< 0.01	2.43	0.124	0.04	0.01	0.925	< 0.01
Stimuli type × Participant gender	1.49	0.227	0.03	0.60	0.443	0.01	0.27	0.603	0.01	0.01	0.946	< 0.01
Stimuli type × Actor gender	0.89	0.350	0.02	0.25	0.617	< 0.01	0.09	0.772	< 0.01	2.34	0.132	0.04
Participant gender × Actor gender	1.67	0.201	0.03	1.72	0.195	0.03	0.89	0.350	0.02	1.94	0.169	0.03
Stimuli type × Participant gender × Actor gender	1.24	0.271	0.02	1.67	0.202	0.03	< 0.01	0.980	< 0.01	0.02	0.876	< 0.01

The results were obtained using repeated-measures ANOVAs with stimulus type (painful vs. non-painful) × participant gender (female vs. male) × actor gender (female vs. male). Significant (*p* < 0.05) differences are indicated in boldface

##### Pain intensity

Pain intensity rating scores were moderated by the main effect of stimulus type [*F*_(1,58)_ = 831.44, *p* < 0.001, η_p_^2^ = 0.96]. Painful action videos (6.31 ± 0.11) were more painful than non-painful action videos (2.24 ± 0.11).

##### Affective valence

Affective valence rating scores were moderated by the main effect of stimulus type [*F*_(1,58)_ = 31.28, *p* < 0.001, η_p_^2^ = 0.35]. Painful action videos (3.88 ± 0.16) were more negative than non-painful action videos (5.01 ± 0.09).

##### Arousal

Arousal rating scores were moderated by the main effect of stimulus type [*F*_(1,58)_ = 52.60, *p* < 0.001, η_p_^2^ = 0.48]. Painful action videos (4.84 ± 0.23) were rated as more exciting than non-painful action videos (3.11 ± 0.20).

##### Dominance

Dominance rating scores were moderated by the main effect stimulus type [*F*_(1,58)_ = 20.19, *p* < 0.001, η_p_^2^ = 0.26]. Painful action videos (5.88 ± 0.25) were rated as more out of control than non-painful action videos (7.02 ± 0.26).

## Empathy for Action Pain Picture Database (EPSS-Action_Picture)

### Methods

#### Development of stimuli

**Fig. 11:**
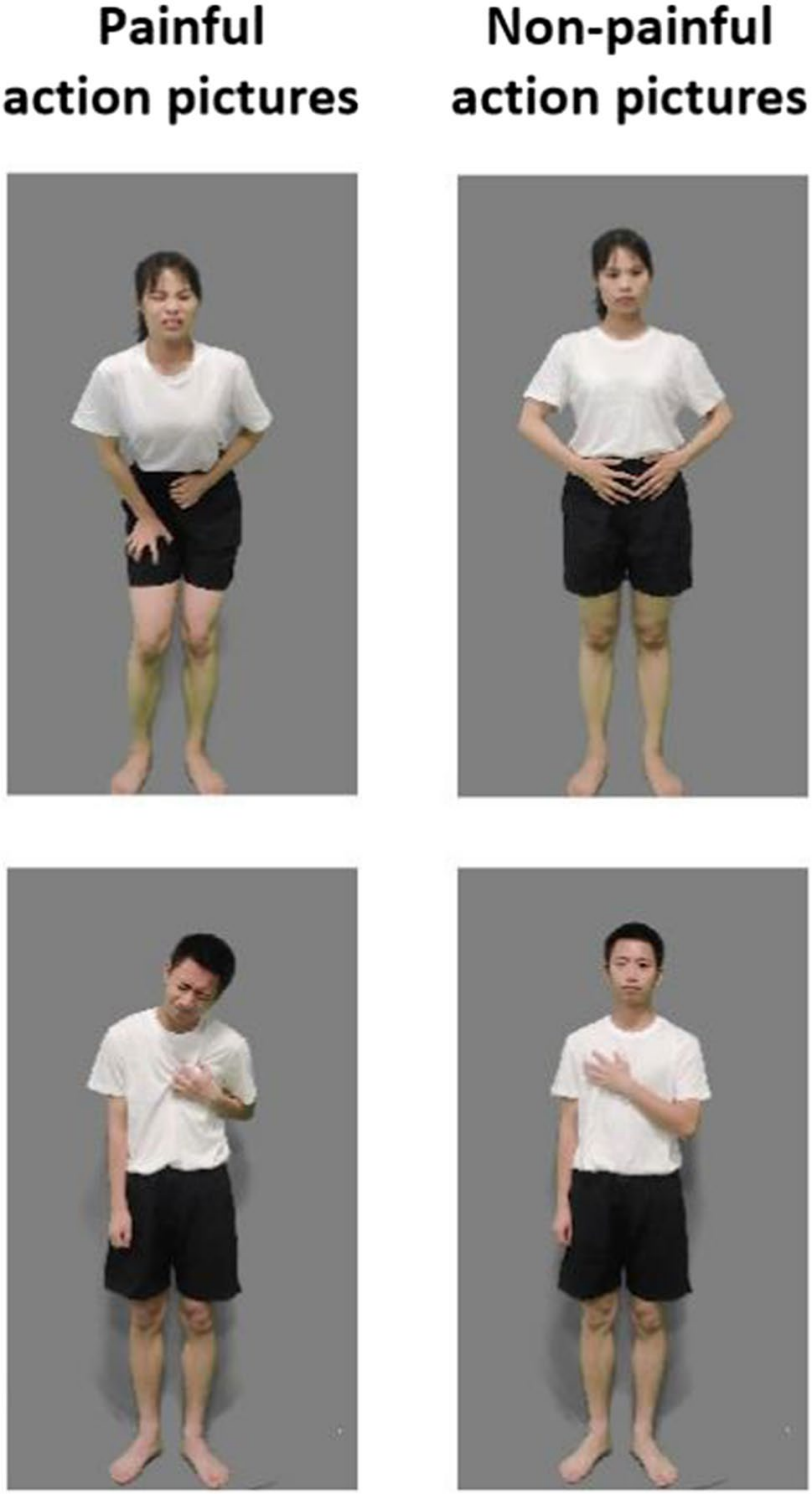
An illustration of painful (*left panel*) and non-painful (right panel) action pictures in the EPSS-Action_Picture

The EPSS-Action_Picture stimuli were developed from the EPSS-Action_Video. A frame of the image best representing the painful or non-painful states of the actor in each video of EPSS-Action_Video was cut out as the stimulus for the EPSS-Action_Picture (examples are shown in Fig. 11). Thus, like the EPSS-Action_Video, the pictures in EPSS-Action_Picture conveyed 12 body parts (head, teeth, neck, arms, elbows, hands, belly, knees, genitalia, chest, waist, and hip). In total, 239 painful and 239 non-painful action pictures from 20 actors (ten females) were selected. The luminance, contrast, and color were matched between the painful and non-painful action pictures. Each picture was 15.24 × 27.9 cm (width × height) and 72 pixels per inch.

#### Validation

##### Participants

Seventy adults (36 females) from Chongqing Normal University, Chongqing, China, were recruited as paid participants. They were aged 18–28 (mean = 22.66, SD = 2.17) and right-handed, with normal or corrected-to-normal vision. The other inclusion criteria were the same as for the EPSS-Limb.

##### Procedures, selection criteria, and statistical analysis

The evaluation procedure, selection criteria of stimuli, participants, and statistical analysis methods for the EPSS-Action_Picture were similar to those for the EPSS-Limb.

### Results

Based on the selection criteria of the stimuli, 478 action pictures (239 painful and 239 non-painful action pictures), recorded by 20 actors (ten females), were selected (illustrated in Fig. 11). Based on the selection criteria of the participants, ten participants (14.3% of the total, six women) were excluded. Thus, rating scores from 60 participants (30 females) aged 18–25 (mean = 21.80, SD = 1.90) were calculated.

#### Characteristics of the EPSS-Action_Picture

The EPSS-Action_Picture provides the descriptive statistics results of the rating scores for the painful and non-painful action pictures across the four dimensions made by the total, female, and male participants (Table 9).

**Table 9:** Descriptive statistics for the EPSS-Action_Picture (mean ± SD)

Dimension	Stimuli type	*N*	Total	Female	Male
Pain intensity	Painful	239	6.09 ± 0.65	6.13 ± 0.68	6.04 ± 0.66
	Non-painful	239	2.13 ± 0.34	1.92 ± 0.37	2.35 ± 0.35
Affective valence	Painful	239	4.00 ± 0.25	3.59 ± 0.36	4.40 ± 0.28
	Non-painful	239	4.89 ± 0.15	5.03 ± 0.19	4.75 ± 0.21
Arousal	Painful	239	4.85 ± 0.40	5.02 ± 0.43	4.68 ± 0.45
	Non-painful	239	2.99 ± 0.24	3.09 ± 0.32	2.89 ± 0.27
Dominance	Painful	239	5.71 ± 0.34	5.86 ± 0.39	5.57 ± 0.37
	Non-painful	239	6.97 ± 0.17	7.35 ± 0.20	6.60 ± 0.24

#### Reliability of the measurements of the EPSS-Action_Picture

The internal consistency of participant assessments of the EPSS-Action_Picture was estimated by calculating split-half reliability scores. The Spearman–Brown formula reliability scores were particularly high in four-dimensional ratings (Pain intensity: *r* = 0.99; Affective valence: *r* = 0.99; Arousal: *r* = 0.99; Dominance: *r* = 0.99). Therefore, dimensional ratings of participant assessments might be considered highly homogeneous in the EPSS-Action_Picture.

#### Relationships between dimensions of the EPSS-Action_Picture

**Fig. 12:**
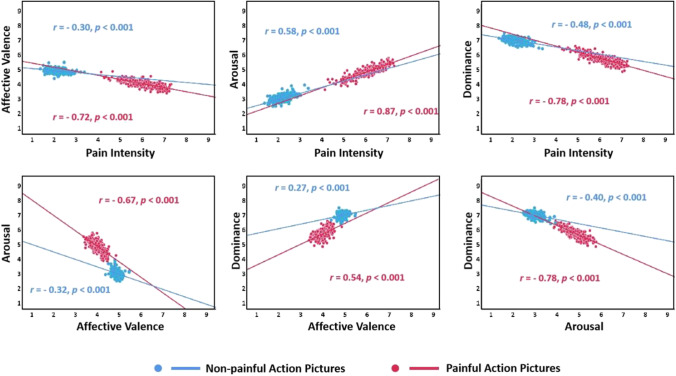
Scatterplot representing the distribution of the mean ratings in four dimensions provided in each painful (*red*) and non-painful (*blue*) action picture of the EPSS-Action_Picture

Figure 12 shows the relationships between the four dimensions of mean rating scores of painful and non-painful action pictures for the EPSS-Action_Picture. Concerning both the painful and non-painful action pictures, significant negative correlations were found in pain intensity × affective valence, pain intensity × dominance, arousal × affective valence, and arousal × dominance. In addition, significant positive correlations were found in pain intensity × arousal and dominance × affective valence (all *p*s < 0.001).

#### Statistical analysis of the EPSS-Action_Picture

Table 10 shows the summary of the statistical analysis of stimulus type × participant gender × actor gender in the participant assessments for four dimensions of the EPSS-Action_Picture.

**Table 10:** Summary of the effect of stimulus type × participant gender × actor gender of the EPSS-Action_Picture

	Pain intensity			Affective valence			Arousal			Dominance		
	*F*	*p*	η_p_^2^	*F*	*p*	η_p_^2^	*F*	*p*	η_p_^2^	*F*	*p*	η_p_^2^
Stimuli type	776.99	**< 0.001**	0.93	21.33	**< 0.001**	0.27	70.20	**< 0.001**	0.55	21.11	**< 0.001**	0.27
Participant gender	1.18	0.283	0.02	2.51	0.119	0.04	0.55	0.462	0.01	1.62	0.208	0.03
Actor gender	0.09	0.772	< 0.01	1.59	0.212	0.03	0.84	0.363	0.01	0.85	0.361	0.01
Stimuli type × Participant gender	3.23	0.078	0.05	7.87	**0.007**	0.12	0.11	0.742	< 0.01	0.70	0.405	0.01
Stimuli type × Actor gender	1.31	0.257	0.02	3.54	0.065	0.06	1.30	0.260	0.02	0.52	0.474	0.01
Participant gender × Actor gender	0.53	0.468	0.01	0.58	0.448	0.01	0.46	0.499	0.01	0.88	0.352	0.02
Stimuli type × Participant gender × Actor gender	3.74	0.058	0.06	0.40	0.528	0.01	7.70	**0.007**	0.18	4.36	**0.041**	0.07

The results were obtained using repeated-measures ANOVAs with stimulus type (painful vs. non-painful) × participant gender (female vs. male) × actor gender (female vs. male). Significant (*p* < 0.05) differences are indicated in boldface

##### Pain intensity

Pain intensity rating scores were moderated by the main effect of stimulus type [*F*_(1,58)_ = 776.99, *p* < 0.001, η_p_^2^ = 0.93]. Painful action pictures (6.09 ± 0.11) were more painful than non-painful action pictures (2.14 ± 0.10).

##### Affective valence

Affective valence rating scores were moderated by the main effect of stimulus type [*F*_(1,58)_ = 21.33, *p* < 0.001, η_p_^2^ = 0.27]. Painful action pictures (4.00 ± 0.16) were more negative than non-painful action pictures (4.89 ± 0.08). The interaction of stimulus type × participant gender [*F*_(1,58)_ = 7.87, *p =* 0.007, η_p_^2^ = 0.12] indicated that for painful action pictures, female participants rated more negative than male participants (male participants: 4.40 ± 0.23, female participants: 3.59 ± 0.23; *p* = 0.017), while no difference was found for non-painful stimuli (male participants: 4.75 ± 0.11, female participants: 5.03 ± 0.11; *p* = 0.205).

##### Arousal

Arousal rating scores were moderated by the main effect of stimulus type [*F*_(1,58)_ = 70.20, *p* < 0.001, η_p_^2^ = 0.55]. Painful action pictures (4.85 ± 0.23) were more exciting than non-painful action pictures (2.99 ± 0.18). The interaction of stimulus type × actor gender × participant gender [*F*_(1,58)_ = 7.70 *p* = 0.007, η_p_^2^ = 0.18] indicated that for the non-painful action pictures, female participants rated pictures with male actors as more exciting than female actors (male actors: 3.12 ± 0.26, female actors: 3.05 ± 0.26; *p* = 0.031), while no other difference was found (*p*s > 0.05).

##### Dominance

Dominance rating scores were moderated by the main effect of stimulus type [*F*_(1,58)_ = 21.11, *p* < 0.001, η_p_^2^ = 0.27]. Painful action pictures (5.71 ± 0.24) were rated as less in control than non-painful action pictures (6.97 ± 0.25). The interaction of stimulus type × actor gender × participant gender [*F*_(1,58)_ = 4.36, *p* = 0.041, η_p_^2^ = 0.07] indicated that for the painful action pictures, female participants rated the pictures with female actors as less in control than male actors (male actors: 5.89 ± 0.33, female actors: 5.82 ± 0.36; *p* = 0.039), while no other difference was found (*p*s > 0.05).

## Discussion

The present study described the generation and assessment of the Empathy for Pain Stimuli System (EPSS), including the Empathy for Limb Pain Picture Database (EPSS-Limb), Empathy for Face Pain Picture Database (EPSS-Face), Empathy for Voice Pain Database (EPSS-Voice), Empathy for Action Pain Video Database (EPSS-Action_Video), and Empathy for Action Pain Picture Database (EPSS-Action_Picture). We believe that these databases, provided for free at https://osf.io/muyah/?view_only=33ecf6c574cc4e2bbbaee775b299c6c1, will be useful for researchers hoping to conduct theoretically motivated explorations of the behavioral, cognitive, and neural processes underlying pain and empathy for pain.

For the stimuli in all the EPSS sub-databases (i.e., EPSS-Limb, EPSS-Face, EPSS-Voice, EPSS-Action_Video, EPSS-Action_Picture), pain intensity was rated using a nine-point Likert scale (1 = *no sensation,* 4 = *pain threshold*, 9 = *the most intense pain imaginable*). The selection criteria for the stimuli were that the pain intensity scores had to be > 4 for the painful stimuli and < 4 for the non-painful stimuli. Thus, the painful and non-painful stimuli could be accurately identified. As a result, the recognition rates for the database were higher than those found for the database of painful stimuli (Belin et al., 2008).

In addition to pain intensity, the other three dimensions (affective valence, arousal, and dominance), which indicated emotional reactions in previous databases (Bai et al., 2005; Lang et al., 1999), were rated. Consistent with a previous database of empathy for pain (Fernandes-Magalhaes et al., 2022), for all the EPSS sub-databases, the painful stimuli were more painful, more arousing, less pleasurable, and less dominant than the non-painful stimuli. The pain intensity scores of painful stimuli for the five sub-databases were negatively correlated with affective valence and dominance. However, they were positively correlated with arousal, suggesting that the more painful the stimuli, the less pleasure, the less dominance, and the more excited people felt.

Consistent with previous studies (Belin et al., 2008), the rating scores of the stimuli in the EPSS were influenced by the gender of the actors in EPSS-Limb, EPSS-Face, and EPSS-Voice. For example, male actors were perceived to feel more pain than female actors in both EPSS-Limb and EPSS-Face. Furthermore, consistent with previous studies on empathy for pain (Fernandes-Magalhaes et al., 2022), the significant interaction effects between actor gender and participant gender were found in affective valence in the EPSS-Limb and EPSS-Face, suggesting that female participants were more sensitive to others’ pain with different genders than male participants. These interactions were also found in the pain intensity, arousal, and dominance of the EPSS-Limb, as well as the pain intensity of the EPSS-Face.

There were some limitations to the EPSS that should be mentioned. First, the models in the EPSS stimuli were Chinese adults, which may be a limitation of this study. In fact, studies have shown that racial bias can be an important factor concerning empathy for pain (Avenanti et al., 2010; Fabi & Leuthold, 2018; Sheng & Han, 2012; Xiang et al., 2018). Therefore, future studies could expand the current database to include models from other races. Second, stimuli in the EPSS were performed by trainee actors or non-professional actors. However, since previous studies have shown that emotional actions produced by professional and non-professional actors are similar (Barliya et al., 2013; Roether et al., 2009) or slightly different (Keefe et al., 2014), the present database could be accepted for the stimuli used in this study, particularly given the strict selection criteria used in this study. Third, for many of the stimuli in the database, the participants recruited in each sub-database had to spend 1–2 h to rate the stimuli during the validation process at a time. Fatigue and practice may have played a role in the rating process. Finally, as the total number of participants included in the EPSS was 350 (70 participants for each sub-database, 312 of whom met the selection criteria) and only young Asian participants were included in the study, a future study would be advisable to expand the validation sample.

In conclusion, we presented the Empathy for Pain Stimuli System (EPSS). The EPSS includes five sub-databases: the Empathy for Limb Pain Picture Database (EPSS-Limb), Empathy for Face Pain Picture Database (EPSS-Face), Empathy for Voice Pain Database (EPSS-Voice), Empathy for Action Pain Video Database (EPSS-Action_Video), and Empathy for Action Pain Picture Database (EPSS-Action_Picture). For each stimulus in the database, the EPSS provides a wealth of information, such as pain intensity, affective valence, arousal, and dominance. We generated and made available this large database concerning empathy for pain, which could be used in behavioral, psychological, and neural research.

### Supplementary information

Below is the link to the electronic supplementary material.

Supplementary file1 (88 KB)
