# Supplementary material for: The Empathy for Pain Stimuli System (EPSS): Development and preliminary validation
Source: Behav Res Methods. 2023 Mar 2;56(2):784–803. doi: 10.3758/s13428-023-02087-4 (PMC10830729; doi:10.3758/s13428-023-02087-4)
Supplement: Supplementary file 1 — Supplementary file1 (88 KB) [file 13428_2023_2087_MOESM1_ESM.docx]

**Appendix_Stimuli used in the training session**

One painful and one non-painful stimuli were used in the training session in five EPSS sub-databases. These stimuli were selected from published paper using similar stimuli as our database but were not included in our database. Stimuli used in the training session of EPSS-Limb, EPSS-Face, EPSS-Voice, and EPSS-Action_Video/EPSS-Action_Picture were selected from Gu, et. al., (2007), Han, et. al., (2009), Meng, et. al., (2020), and Li, et. al., (2022), respectively (see Figure 1_Appendix). In the training session, participants can practice repeatedly until they are familiar with the experimental procedure.


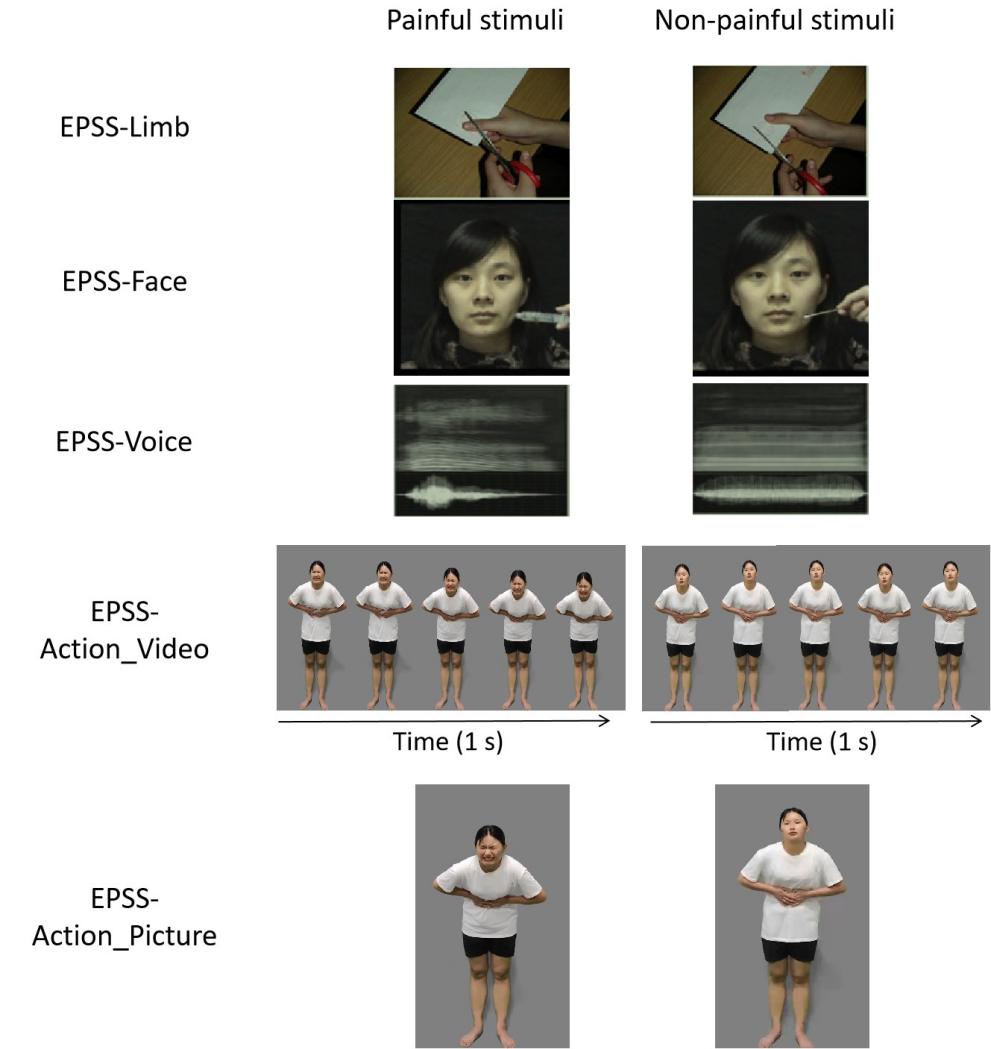


Figure 1_Appendix. Stimuli used in the training session of EPSS sub-databases. EPSS-Limb: Gu et. al., (2007), EPSS-Face: Han et. al., (2009) and Xu et. al., (2009), EPSS-Voice: Meng et. al., (2020), EPSS-Action_Video: Li et. al., (2022), EPSS-Action_Picture: Li et. al., (2022).

**References:**

Gu, X. S., & Han, S. H. (2007). Attention and reality constraints on the neural processes of empathy for pain. *Neuroimage*, *36*(1), 256-267. <https://doi.org/10.1016/j.neuroimage.2007.02.025>

Han, S. H., Fan, Y., Xu, X. J., Qin, J. G., Wu, B., Wang, X. Y., . . . Lihua Mao, L. H. (2009). Empathic neural responses to others' pain are modulated by emotional contexts. *Human Brain Mapping*, *30*, 3227-3237.

Li, Y., Wei, Z., Shao, M., Hong, M., Yang, D., Luo, L., & Meng, J. (2022). Empathy for pain in individuals with autistic traits during observation of static and dynamic stimuli. *Frontiers in Psychiatry*, *13*, 1022087. <https://doi.org/10.3389/fpsyt.2022.1022087>

Meng, J., Li, Z., & Shen, L. (2020). Altered neuronal habituation to hearing others’ pain in adults with autistic traits. *Scientific Reports*, *10*(1), 15019. <https://doi.org/10.1038/s41598-020-72217-x>
